# Erratum for Gaume et al., “Enterovirus A71 crosses a human blood–brain barrier model through infected immune cells”

**DOI:** 10.1128/spectrum.02167-24

**Published:** 2024-09-30

**Authors:** Léa Gaume, Hélène Chabrolles, Maxime Bisseux, Igor Lopes Coqueiro, Lucie Dehouck, Audrey Mirand, Cécile Henquell, Fabien Gosselet, Christine Archimbaud, Jean-Luc Bailly

## Erratum

Volume 12, no. 6, e00690-24, 2024, https://doi.org/10.1128/spectrum.00690-24. Page 1, byline: Igor Lopes Coqueiro’s name should appear as shown in this Erratum.

