# Supplementary material for: Enterovirus A71 crosses a human blood–brain barrier model through infected immune cells
Source: Microbiol Spectr. 2024 May 16;12(6):e00690-24. doi: 10.1128/spectrum.00690-24 (PMC11237604; doi:10.1128/spectrum.00690-24)
Supplement: Supplemental material — Fig. S1-S8; Tables S1 and S2. [file spectrum.00690-24-s0001.pdf]

Supplemental Material

Enterovirus A71 crosses a human blood-brain barrier model through infected immune cells

Léa Gaume, Hélène Chabrolles, Maxime Bisseux, Igor Lopez-Coqueiro, Lucie Dehouck, Audrey Mirand, Cécile Henquell, Fabien Gosselet, Christine Archimbaud, Jean-Luc Bailly

SUPPLEMENTARY METHODS 2

Virus strains used in the study

Virus titration assays

Efflux activity of the adenosine triphosphate-binding cassette (ABC) transporters

Cell viability assays

Western Blot

Assessment of cellular mRNA expression with RT-qPCR assays

SUPPLEMENTARY RESULTS 4

Susceptibility of undifferentiated CD34+-derived endothelial cells to EV-A71

Expression of zonula-occludens 1 and claudin-5 proteins in EV-A71-infected hBLECs

Efflux activity of ABC transporters in EV-A71-infected hBLECs

REFERENCES 6

Table S1. Characteristics of enterovirus isolates used in the study 7

Table S2. Antibodies used in flow cytometry experiments 7

Figure S1. Susceptibility of undifferentiated CD34+-derived endothelial cells to enterovirus A71 (EV-A71) 9

Figure S2. **Susceptibility of RD cells to enterovirus A71 (EV-A71)** 11

Figure S3**.** Barrier properties of the mock-infected hBLEC model 12

Figure S4. Expression of zonula occludens 1 (ZO-1) and claudin-5 (CLDN-5) proteins in EV-A71-infected hBLECs 12

Figure S5. Efflux activity of ABC transporters in EV-A71-infected hBLECs 14

Figure S6. Kinetics of permeability and infectious viral particles after Echo-6 infection 14

Figure S7. White blood cells transmigrating through hBLECs on transwell insert 15

Figure S8. EV-A71-infected leukocytes detection on the upper compartment of the in vitro human BBB. 15

SUPPLEMENTARY METHODS

Virus strains used in the study

Three clinical isolates of EV-A71 designated C1-06, C1-16, and C4, and one isolate of echovirus 6 (Echo-6) were used in this study (**Table S1**). The viruses were obtained from the virology laboratory of the University Hospital of Clermont-Ferrand (France). The virus strains were propagated in the rhabdomyosarcoma (RD) cell line obtained from the European Collection of Authenticated Cell Culture (Cat. No. 8511502). The RD cells, used at passages between 44 and 80, were cultured in DMEM high glucose (Dutscher) containing 2 mM glutamine, and supplemented with 10% fetal bovine serum (FBS; Eurobio, #CVFSVF00-0U) and 1% of streptomycin-penicillin. The virus stocks used throughout the study were checked by sequencing the complete viral genomes with a high-throughput sequencing method (data not shown) and titrated as described below.

Virus titration assays

The concentration of infectious virus particles in infected hBLECs was determined by a titration assay based on the limiting dilution method previously reported (1) (**Bailly et al., 1991**). Each sample was subjected to appropriate 10-fold dilutions. A series of eight 4-fold dilutions was performed from the last 10-fold dilution, and 50 µL aliquots of each 4-fold dilution were transferred to 11 wells of a 96-well cell culture plate. Negative controls were included in each titration plate. All the wells were filled with 150 µL of a RD cell suspension prepared at 80,000 cells/mL in 5% FBS DMEM. The plates were incubated at 37°C for seven days. Each well was scored for a cytopathic effect and the number of infectious particles was calculated using an in-house algorithm and expressed as the most probable number of infectious units (MPNCU) (1) (**Bailly et al., 1991**).

Efflux activity of the adenosine triphosphate-binding cassette (ABC) transporters

The efflux activity of P-glycoprotein (P-gp or ABCB1) and breast cancer resistance protein (BCRP or ABCG2) was assessed by testing the intracellular accumulation of rhodamine 123 (R123, Merck Sigma), a fluorescent tracer dye, with and without elacridar, an inhibitor of ABC

transporters (Merck, Sigma) as previously assessed (2, 3) (**Sevin et al., 2019; Versele et al., 2020**). Pericytes were scrapped from 10-day co-cultures and the Transwell inserts were placed in a 12-well plate filled with HBSS (1.5 mL per well). hBLECs in the upper compartment were exposed for 2 h at 37°C to a 5 µM R123 solution or a solution containing R123 (5 µM) and elacridar (0.5 µM). After incubation, the solutions were removed and hBLECs were extensively washed five times with ice-cold complete HBSS. The cells were lysed with 300 µL of cold RIPA buffer (Merck, Sigma; #R0278). Aliquots of each sample were transferred to black 96-well plates and R123 fluorescence was determined with a Fluoroscan analyzer (Thermo Scientific) at excitation/emission wavelengths of 501/538 nm. The mean fluorescence was calculated from three independent replicates.

Cell viability assays

The MultiTox-Fluor Multiplex Cytotoxicity Assay (Promega) was used to assess the viability of infected ECs. Living cells were quantified by the detection of a fluorogenic, cell-permeant peptide substrate (glycyl-phenylalanyl-aminofluorocoumarin, GF-AFC). This substrate is cleaved by the live-cell protease activity to release AFC, which can be detected by fluorescence. CD34+-derived ECs were grown in black 96-well plates, one plate for each time point tested (24, 48, and 72 h pi). The infected cell monolayers were assayed with 100 µL of the reagent and fluorescence was measured after 1.5 h at excitation/emission wavelengths of 400/505 nm.

Western Blot

The infected hBLEC samples were lysed in the RIPA buffer, 20 mM Tris-HCl pH 7.4, 150 mM NaCl, 2 mM EDTA, 1% NP-40, 20 mM Na_3VO_4, 10 mM NaF, and a protease inhibitor cocktail (Roche, #04693116001). After cell disruption and centrifugation at 10,000 g at 4°C for 10 min, the supernatants were collected and proteins were quantified with the DC Protein Assay (Bio-Rad). Laemmli buffer was added to the protein extracts (1:4) and incubated for 5 min at 95°C. Protein extracts were separated in a 4-15% Mini-Protean TGX precast gel (Bio-Rad #456-1084) and transferred to nitrocellulose membrane (Trans-Blot® Turbo™ Transfert System, Bio-Rad). The unspecific binding of antibodies to non-target structures was blocked with 0.1% Tween 20 and 5% BSA in PBS: primary antibodies were included in the blocking buffer. The primary antibodies used were rabbit monoclonal anti-ZO-1 (Cell Signaling D6L1E #13663, 1:1000),

rabbit polyclonal anti-CLDN-5 (Thermo Fisher #34-1600, 1:1000), and rabbit anti-β-actin (Cell signaling 13E5 #4970, 1:1000). After an incubation at 4°C overnight and three washes with 0.1% Tween 20 in PBS, the membranes were incubated for 1 h with an anti-rabbit horseradish peroxidase-conjugated secondary and with Clarity™ Western ECL substrate (#1705060, Bio-Rad). ZO-1 and CLDN-5 protein expression was quantified relative to β-actin by assessing band intensities (n = 6 replicates) using Image Lab software 6.1.0 (Bio-Rad Laboratories Inc.).

Assessment of cellular mRNA expression with RT-qPCR assays

Total nucleic acids were extracted from infected hBLEC samples as described above. The nucleic acid extract (50 µL) was treated with 10 µL of DNase I solution for 10 min at 37°C, and with RNAClean XP (Beckman Coulter). cDNA was synthetized from total RNA using random hexamers and SuperScript IV Kit, for 10 min at 23°C, 10 min at 50°C, and 10 min at 80°C, as recommended by the manufacturer. Real-time PCR amplification was performed with the ABsolute qPCR Mix, SYBR Green, ROX kit (ThermoScientific™) and a CFX Opus 96 Real-Time PCR System. Primers for each gene were as follows: ZO-1 (Fwd: 5'-CTCATTTTCAGAGTGGGGAAAC-3'; Rev: 5'-GGTCATTTTCCTGTAGCTGTCC-3'), CLDN-5 (Fwd: 5'-GAGGCGTGCTCTACCTGTTTT-3'; Rev: 5'- CACAGACGGGTCGTAAAACTC-3'), and β-actin (Fwd: 5'-TCGTGCGTGACATTAAGGAG-3'; Rev: 5'-AGCACTGTGTTGGCGTACAG-3'). The PCR conditions used were the following: one cycle of 15 min at 95°C, 40 cycles of 15 s at 95°C, 30 s at 50.7 °C (ZO-1), 60 °C (CLDN-5), and 58 °C (β-actin), and 30 s at 72°C. Gene expression levels were assessed by the $\Delta\Delta$Ct method and normalized to the β-actin reference gene.

SUPPLEMENTARY RESULTS

Susceptibility of undifferentiated CD34+-derived endothelial cells to EV-A71

We explored the susceptibility of undifferentiated CD34+-derived ECs before investigating EV-A71 replication in hBLECs and BBB crossing mechanism (Fig. S1). In comparison to microvascular brain ECs, the undifferentiated CD34+-derived ECs had features of peripheral ECs, such as cobblestone-like morphology, expression of high levels of CD31, VE-cadherin, and vWF, and the ability to incorporate acetyl low density lipoprotein (4) (**Cecchelli et al., 2014**). The percentage of infected cells was determined at 6 and 24 h post-infection (hpi) (Fig. S1B). At 6 hpi, 1% to 2% of ECs were infected and at 24 hpi, the proportions of infected cells were

as follows for the 3 EV-A71 strains: C4, 1.7% (range, 0% – 4%), C1-16, 2.7% (range 1.4% – 4.2%), and C1-06, 3.1% (range 0% – 7%). The virus yields increased for all EV-A71 strains at 24 hpi over the time 6 hpi: there was a 21-fold increase for the C4 virus, and a 4-fold increase for the other EV-A71 strains (Fig. S1C). The viability of CD34+-derived ECs was moderately affected by EV-A71 infection at 24 and 48 hpi (Fig. S1D). Overall, the undifferentiated CD34+-derived ECs were moderately susceptible to EV-A71.

Expression of zonula-occludens 1 and claudin-5 proteins in EV-A71-infected hBLECs

We looked at whether virus infection could impair ZO-1 and CLDN-5 proteins, selected as two cellular components involved in or associated with the barrier features. For this purpose, the levels of ZO-1 and CLDN-5 were assessed after inoculation with the C1-16 and C1-06 strains (MOI = 5) and compared with the levels determined in mock-infected controls (Fig. S3). The mRNA levels of ZO-1 and CLDN-5 (normalized to the β-actin levels) in infected hBLECs at 24 and 48 hpi showed no difference compared with mock-infected controls (Fig. S3A and S3B). The levels of ZO-1 and CLDN-5 proteins were analyzed by western blot at the same times pi (Fig. S3C) and subjected to densitometric analysis (Fig. S3D and S3E). The normalized protein levels of ZO-1 showed no variation within the infected hBLECs at 24 and 48 hpi *versus* mock-infected controls. Compared with mock-infected controls, the C1-16-infected hBLECs had a mean variation of 101% (range, 82% – 121%), and the C1-06-infected hBLECs of 105% (range, 73% – 153%). For both viruses, the levels of CLDN-5 protein at 24 hpi showed no significant variation in the infected hBLECs *versus* mock-infected controls (C1-16 [mean variation, 97%; range 57% – 136%], C1-06 [mean, 124%; range 82% – 223%]) (Fig. S3E). At 48 hpi, CLDN-5 protein level was barely reduced by 1.2-fold in the C1-16-infected hBLECs (mean, 81%; range 45% – 118%) and by 1.5-fold in C1-06-infected hBLECs (mean, 80.5%; range 69% – 92%). Localization of ZO-1 and CLDN-5 proteins was analyzed at 24 and 48 hpi by confocal microscopy after IF staining in infected and non-infected hBLECs (Fig. S3F). A continued localization of ZO-1 and CLDN-5 proteins was observed at the periphery of non-infected controls and infected hBLECs at 24 and 48 hpi. Overall, the data indicate no variation in the levels and localization of CDLN-5 and ZO-1 proteins in EV-A71-infected hBLECs.

Efflux activity of ABC transporters in EV-A71-infected hBLECs

In the light of the above findings, we looked at whether virus infection could impair a characteristic function of hBLECs not involved in the barrier properties. For this experiment, we chose the ABC efflux transporters expressed by brain microvascular ECs, which are considered to be CNS gatekeepers because they ensure correct brain functioning. The efflux activity of ABC transporters was assessed by analyzing the intracellular accumulation of the fluorescent R123 compound in hBLECs at 48 h pi with or without elacridar, an efflux pump inhibitor (Fig. S4). First, to investigate whether the ABC transporters were present and functional, we assessed the percentage of R123 accumulation in the absence or presence of the efflux pump inhibitor and determined the difference (Fig. S4A). In mock-infected hBLECs, an increase in R123 accumulation is measured in the presence of the efflux pump inhibitor, showing a mean increase of 32.2% (range, 4.4% – 54.2%). Similar increases were observed for infected conditions (MOI 5), showing a mean increase of 22.4% (range, 9.3% – 33.9%) for C1-16, and a mean increase of 26.5% (range, 13% – 40.8%) for C1-06. These data indicate that ABC transporters were present and functional in mock-infected and infected hBLECs. We then investigated the functional properties of ABC transporters in hBLECs infected with C1-16 or C1-06 (Fig. S4B). Accordingly, the intracellular accumulation of R123 in infected hBLECs was analyzed by setting at 100% the R123 levels in mock-infected hBLECs. Compared with these controls, the R123 levels in infected hBLECs were lower: 81.3% (range, 70.1% – 93.9%) for C1-06, and 90% (range, 73.3% – 106.6%) for C1-16. Overall, the data indicate that although the ABC transporters are efficiently inhibited by elacridar in EV-A71-infected hBLECs, R123 was released in higher amounts than in uninfected cells. This suggests that efflux activity was enhanced in infected hBLECs.

REFERENCES

(1) Bailly JL, Chambon M, Peigue-Lafeuille H, Laveran H, De Champs C, Beytout D. Activity of glutaraldehyde at low concentrations (less than 2%) against poliovirus and its relevance to gastrointestinal endoscope disinfection procedures. Appl Environ Microbiol. 1991; 57(4):1156-60. doi: 10.1128/aem.57.4.1156-1160.1991.

(2) Sevin E, Dehouck L, Versele R, Culot M, Gosselet F. A Miniaturized Pump Out Method for Characterizing Molecule Interaction with ABC Transporters. Int J Mol Sci. 2019 Nov 6;20(22):5529. doi: 10.3390/ijms20225529. PMID: 31698745; PMCID: PMC6888615.

(3) Versele R, Corsi M, Fuso A, Sevin E, Businaro R, Gosselet F, Fenart L, Candela P. Ketone Bodies Promote Amyloid-β₁₋₄₀ Clearance in a Human in Vitro Blood-Brain Barrier Model. Int J Mol Sci. 2020 Jan 31;21(3):934. doi: 10.3390/ijms21030934. PMID: 32023814; PMCID: PMC7037612.

(4) Cecchelli, R, Aday, S, Sevin, E, Almeida, C, Culot, M, Dehouck, L, et al. (2014). A stable and reproducible human blood-brain barrier model derived from hematopoietic stem cells. PLoS One. 9, e99733. doi: 10.1371/journal.pone.0099733.

Table S1. Characteristics of enterovirus isolates used in the study.

Virus designation in the present study	Phylogenetic assignment (type / subgenogroup)	Collection year of the initial clinical sample	Initial clinical specimen	Main clinical manifestations	Designation of virus isolate	GenBank accession number[a]	Infectious titer of virus stock used in the study (MCPNU/mL)
C1-06	EV-A71/C1	2006	Throat	Asymptomatic	CF2006/210042	LR027547<br>HG934219	9.8×10^6
C1-16	EV-A71/C1-like	2016	Throat	Mild HFMD	CF316250102	LR027524	38×10^6
C4	EV-A71/C4	2004	Stool	HFMD with meningitis (positive CSF)	CF2004/192013	HG934208	20×10^6
Echo-6	Echovirus 6	2001	Stool	Meningitis (positive CSF)	CF2660-01	FN688519<br>FN688558	576×10^6

[a]The accession number indicated is for the nucleotide sequence of the P1 genomic region. The virus suspensions used in the study were controlled by sequencing the complete genomes.

Table S2. Antibodies used in flow cytometry experiments.

Antibody	Species	Company (Catalog#)
Anti-human CD45 – V500	Mouse	BD Biosciences #560777
Anti-human CD15 – V450	Mouse	BD Biosciences #561584
Anti-human CD14 – APC-H7	Mouse	BD Biosciences #641394
Anti-human CD19 – Pe-Cy7	Mouse	BD Biosciences #557835

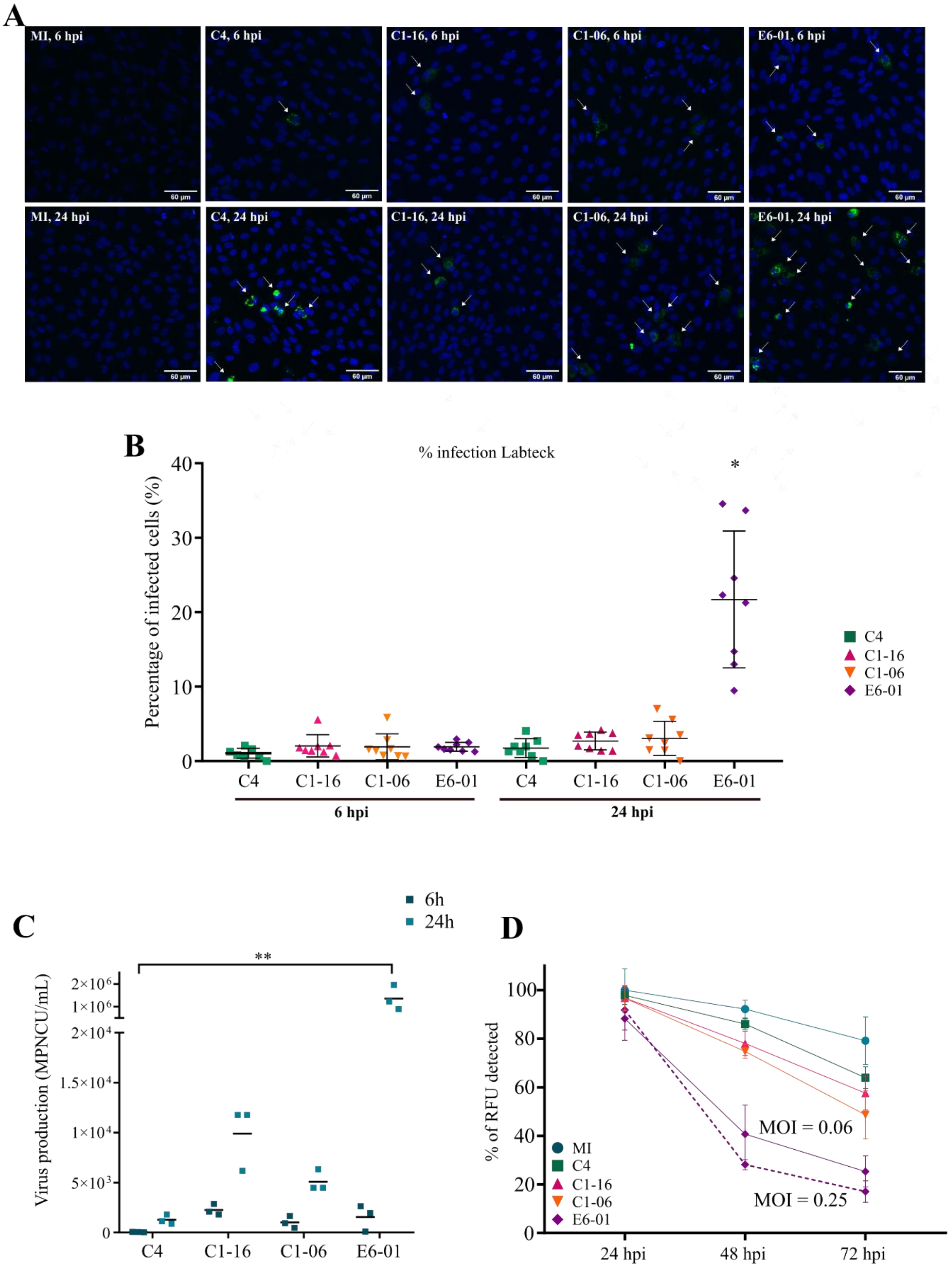

Figure S1. Susceptibility of undifferentiated CD34+-derived endothelial cells to enterovirus A71 (EV-A71). (A) The undifferentiated CD34+-derived endothelial cells grown on Matrigel-coated glass slides were inoculated (MOI = 1) with the EV-A71 strains C4, C1-16 and C1-06, and the cytolytic control Echo-6. Mock-infected (MI) condition was used as control. The presence of intracellular viral replicative dsRNAs was detected by immunofluorescence microscopy at 6- and 24-hours post-infection (hpi) (green color and arrow). Nuclei were

counterstained with DAPI (blue). **(B)** Infected undifferentiated CD34+-derived endothelial cells expressed as the percentage of cells showing immunofluorescence staining of replicative dsRNAs. **(C)** Virus production at 6 and 24 h pi was determined as the most probable number of infectious viruses per mL of supernatant. **(D)** Living cells were quantified by the detection of a fluorogenic, cell-permeant peptide substrate (glycyl-phenylalanyl-aminofluorocoumarin). The relative fluorescence units (RFU) were determined in mock-infected cells (blue circles) and in cells infected (MOI = 1) by each EV-A71 strain: C4 (green), C1-16 (pink), and C1-06 (orange). For the cytolytic Echo-6 virus (purple), inoculation was done at MOI = 0.25 (dotted line) and MOI = 0.06 (full line). The result for mock-infected cells was set at 100%. The data are represented at the mean ± standard deviation obtained from three independent experiments. Statistical significance was determined with Kruskal and Wallis test. *P ≤ 0.05; **P ≤ 0.01.

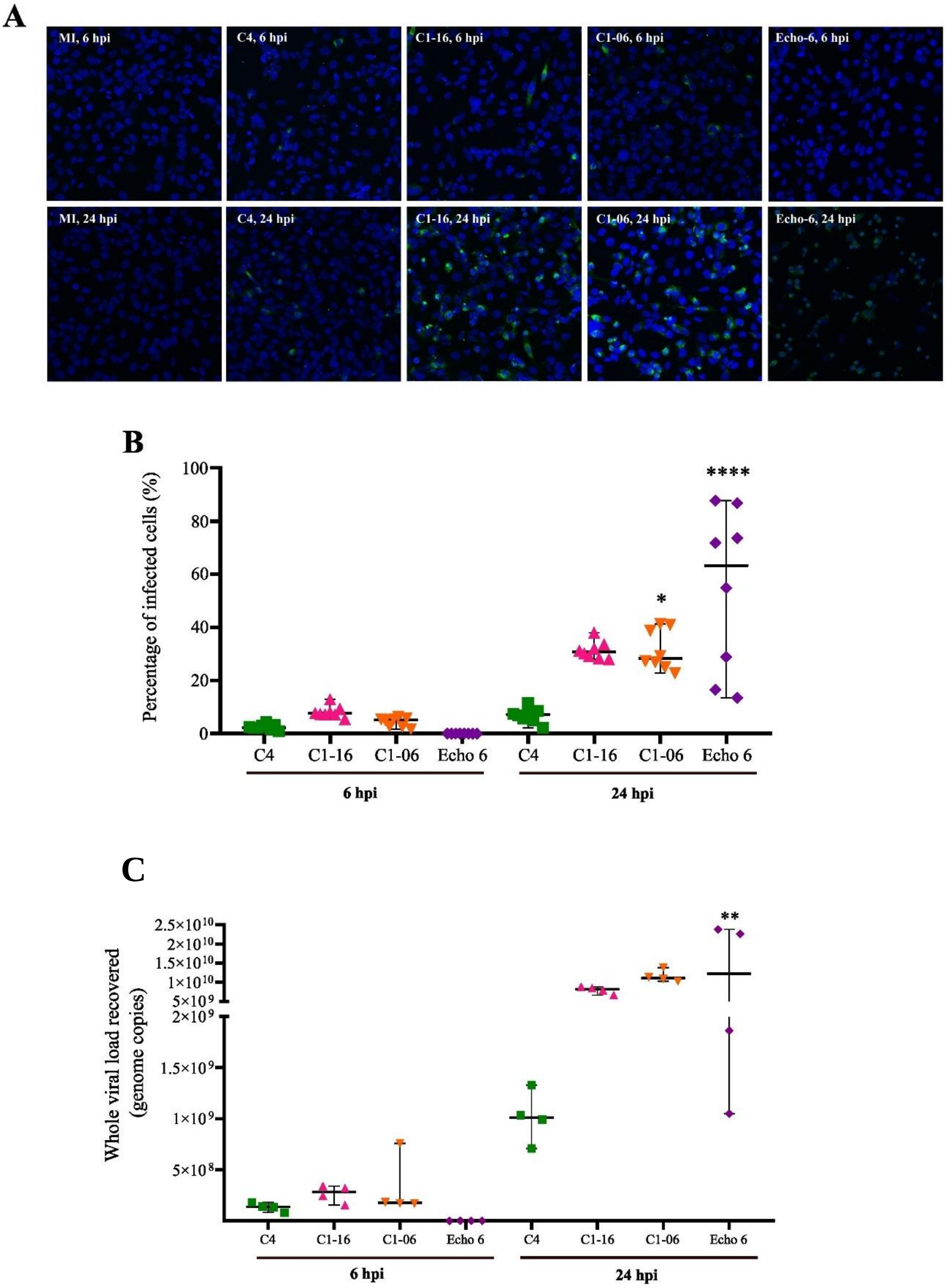

Figure S2. Susceptibility of RD cells to enterovirus A71 (EV-A71). (A) RD cells grown on glass slides were inoculated (MOI = 1) with the EV-A71 strains C4, C1-16 and C1-06, and the cytolytic control Echo-6. Mock-infected (MI) condition was used as control. The presence of intracellular viral replicative dsRNAs was detected by immunofluorescence microscopy at 6- and 24-hours post-infection (hpi) (green color). Nuclei were counterstained with DAPI (blue). **(B)** The infected RD cells were determined as the percentage of cells showing immunofluorescence staining for replicative dsRNAs. **(C)** The viral RNAs within RD cells were

detected by RT-qPCR at 6 and 24 h pi, and results were expressed as total genome copies. The data are represented at the median ± interquartile range deviation obtained from two independent experiments. Statistical significance was determined with Kruskal and Wallis test. **P ≤ 0.01, **** P≤ 0.0001 .

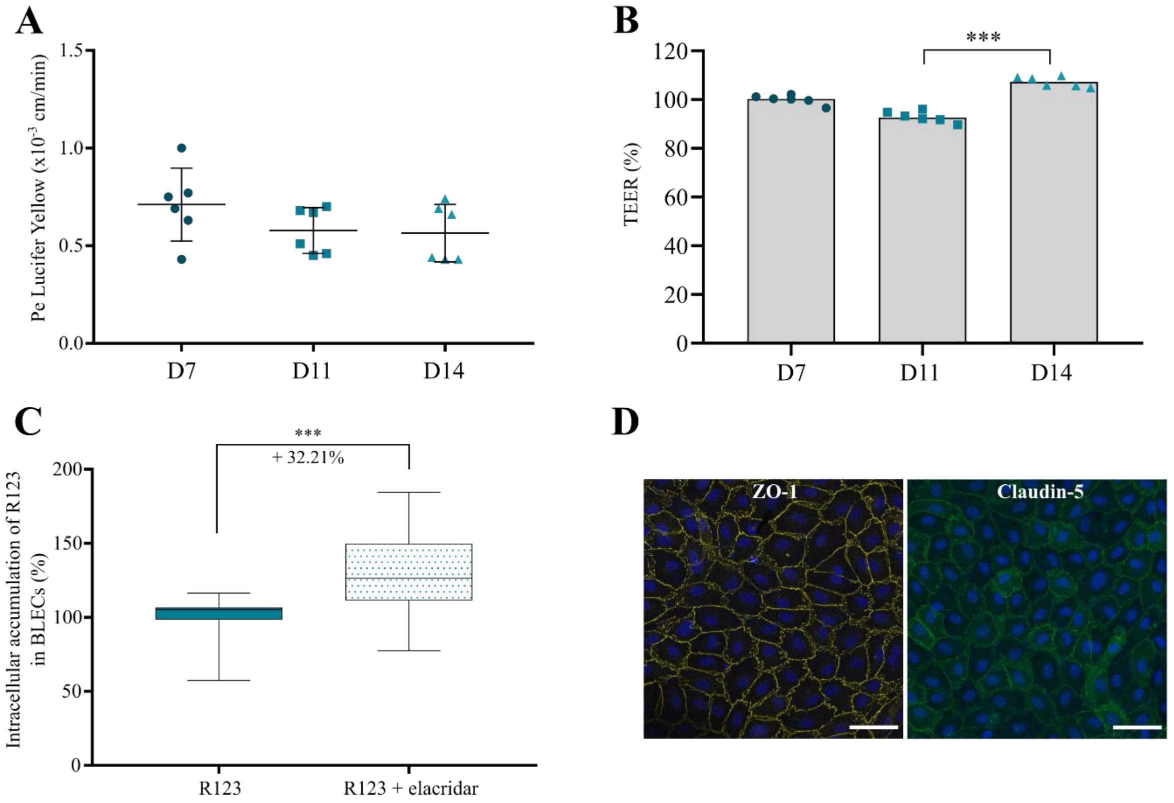

Figure S3. Barrier properties of the mock-infected hBLEC model. (**A**) Paracellular permeability of Lucifer Yellow at 7, 11 and 14 days after model building. (**B**) Transendothelial electrical resistance (TEER) realized at 7, 11 and 14 days after model building. The percentage of TEER was calculated by using the following formula: percentage of TEER = (TEER at D7)/(TEER at D11 or D14)×100%. (**C**) The efflux activity of ABC transporters was assessed by measuring the intracellular accumulation of rhodamine 123 (R123) in the absence (full box) and presence (hatched box) of elacridar, an inhibitor of transporters. The intracellular levels of R123, in the absence of elacridar was set at 100%. (**D**) Immunofluorescence and confocal microscopy of mock-infected (MI) BLECs for ZO-1 and CLDN-5. Statistical significance was determined with Kruskal and Wallis test. **P ≤ 0.01; ***P ≤ 0.001.

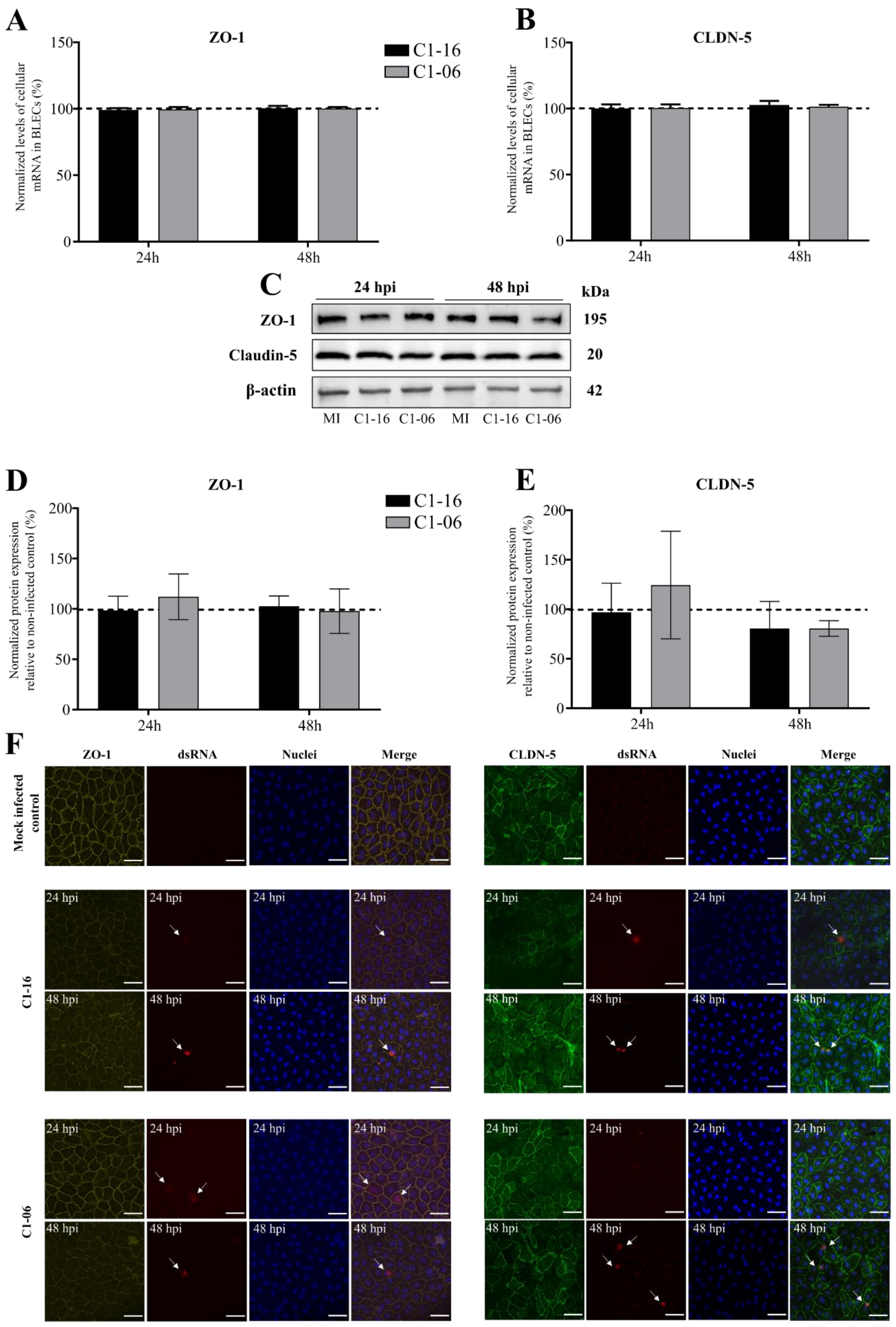

Figure S4. Expression of zonula occludens 1 (ZO-1) and claudin-5 (CLDN-5) proteins in EV-A71-infected hBLECs. hBLECs were infected (MOI = 5) with the EV-A71 strains C1-16 and C1-06 and analyzed at the indicated times pi. The levels of ZO-1 (**A**) and CLDN-5 (**B**) mRNA in the

infected BLECs were normalized by the β-actin signals. The values are expressed as percentage of mock-infected controls. (**C**) Immunoblot analyses of the expression of ZO-1 and CLDN-5 in mock- and EV-A71-infected hBLECs at the indicated times pi. Representative images are shown. Expression of ZO-1 (**D**) and CLDN-5 (**E**) proteins in hBLECs were normalized by β-actin signals. The values are expressed as percentage of mock-infected controls. (**F**) Immunofluorescence and confocal microscopy of mock-infected (MI) and EV-A71-infected BLECs for ZO-1 (yellow), CLDN-5 (green), and viral dsRNA (red) replication intermediates. P-value determined with one-way ANOVA with multiple comparison test, not significant.

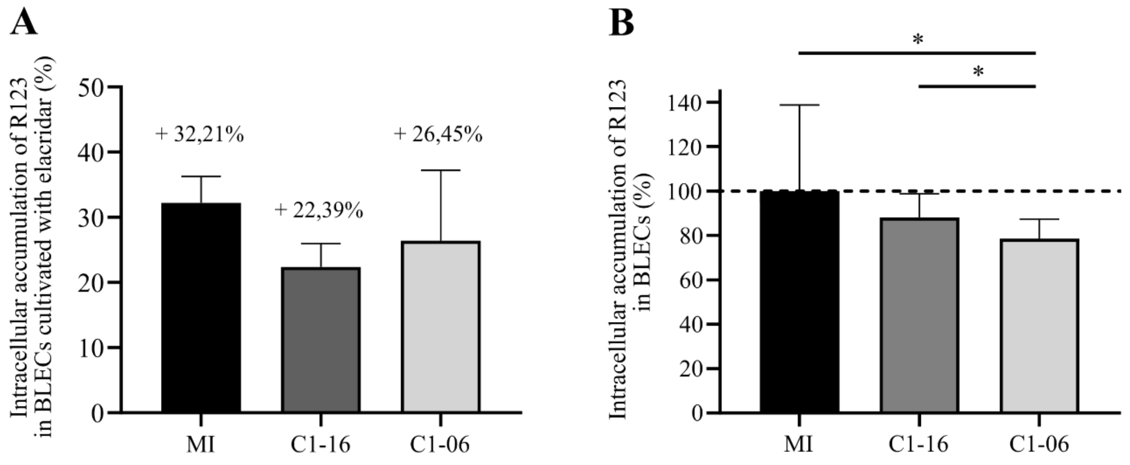

Figure S5. Efflux activity of ABC transporters in EV-A71-infected hBLECs. The efflux activity of ABC transporters was assessed by measuring the intracellular accumulation of the fluorescent compound rhodamine 123 (R123) in the absence or presence of elacridar, an efflux pump inhibitor. hBLECs were mock-infected or infected at MOI = 5 with the EV-A71 C1-16 and C1-06 strains (**A**). The intracellular accumulation of R123 in EV-A71-infected hBLECs was analyzed by setting the intracellular levels of R123 in mock-infected hBLECs at 100%. Statistical analysis shows a difference between the mock-infected controls and the infected conditions (**B**). The data are represented as the mean ± standard deviation obtained from triplicate measurements of six independent replicates. Statistical significance was determined with one-way ANOVA with multiple comparison test (**A**) and Kruskal and Wallis test (**B**). *P ≤ 0.05.

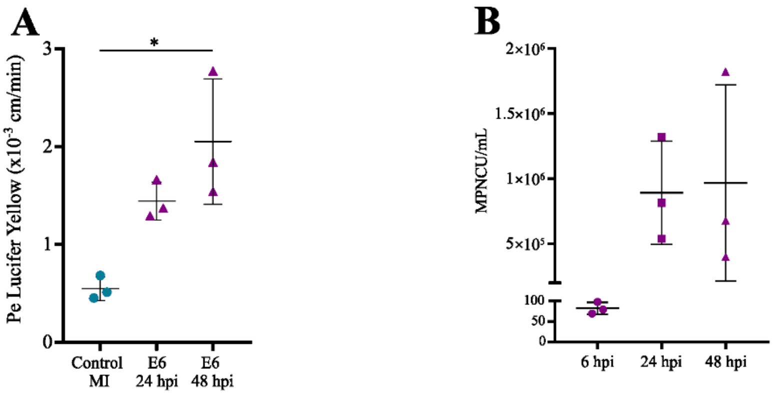

Figure S6. Kinetics of permeability and infectious viral production after Echo-6 infection. (**A**) Permeability coefficient (Pe) of Lucifer Yellow determined after inoculation of hBLECs (MOI =

1) by the Echo-6 at 24 and 48 hpi. MI, mock-infected hBLECs. The data are the means of three independent replicates, with the error bars representing standard deviation. (**B**) Viral production determined in hBLECs infected with Echo-6 strain by virus titration. The data are expressed as the most probable number of infectious units produced per 24 hours at 6, 24, and 48 hpi. The data are the means of three replicates with the error bar representing the standard deviation. Statistical significance was determined with Kruskal and Wallis test. *P ≤ 0.05.

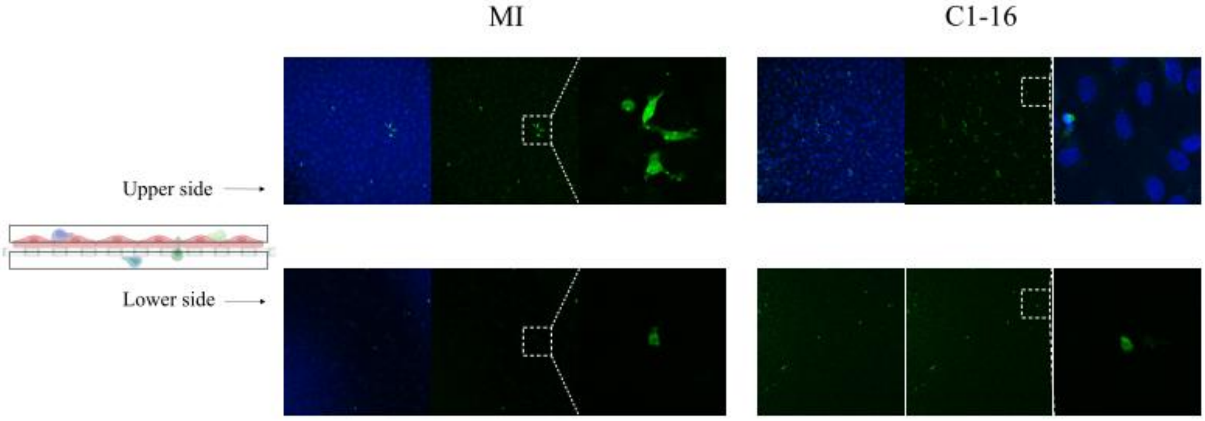

Figure S7. White blood cells transmigrating through hBLECs on transwell insert. Image acquisitions of the upper (luminal) and lower (basolateral) sides of hBLECs were obtained by confocal microscopy. Transmigrating immune cells labelled with CytoTracker (green) were visualized at the upper and lower sides. hBLECs nuclei are in blue.

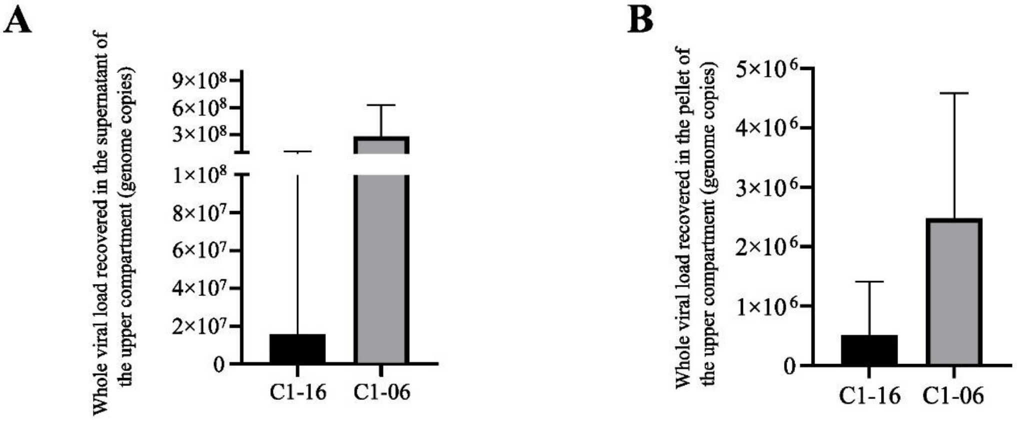

Figure S8. Detection of EV-A71-infected leukocytes on the upper compartment of the *in vitro* human BBB. After transmigration, leukocytes in the upper transwell compartment were centrifuged. RT-qPCR was performed to detect EV-A71 viral RNA in the medium (**A**) and in the pellet of leukocyte cells (**B**). Data are expressed as the median ± interquartile range. An unpaired t test was performed, but no significant variations were measured.
